# Robot-Assisted Versus Laparoscopic Ureteroureterostomy for Duplicated Kidney Malformations in Infants: A Comparative Cohort Study

**DOI:** 10.3390/children13060839

**Published:** 2026-06-22

**Authors:** Huazhang Liu, Minghui Pan, Liming Jin, Guangjie Chen, Chang Tao, Xiang Yan

**Affiliations:** Department of Urology, Children’s Hospital, Zhejiang University School of Medicine, National Clinical Research Center for Children and Adolescents’ Health and Diseases, No. 3333 of Binsheng Road, Hangzhou 310003, China; 6516146@zju.edu.cn (H.L.);

**Keywords:** laparoscopic ureteroureterostomy, duplicated kidney, minimally invasive surgery, postoperative outcomes

## Abstract

**Highlights:**

**What are the main findings?**

- Both robotic and laparoscopic ureteroureterostomy achieved favorable safety and efficacy outcomes in infants with duplicated kidney malformations.
- Robot-assisted laparoscopic ureteroureterostomy reduced operative time, while postoperative recovery and follow-up outcomes were comparable between the two approaches.

**What are the implications of the main findings?**

- This study provides direct clinical evidence for surgical decision-making between robot-assisted and laparoscopic ureteroureterostomy in infants with duplicated kidney malformations.
- These findings provide a basis for future multicenter prospective studies aimed at optimizing surgical selection and clinical management strategies.

**Abstract:**

**Objective:** The aim of this study was to evaluate the safety and efficacy of robot-assisted laparoscopic ureteroureterostomy (RALUU) and laparoscopic ureteroureterostomy (LUU) for duplicated kidney malformations in infants. **Methods:** This retrospective comparative cohort included infants with duplicated kidney malformations who underwent RALUU or LUU between May 2021 and April 2025. Perioperative variables assessed included operative duration, blood loss, oral feeding time, FLACC pain score, hospital stay, and complications. Follow-up outcomes included changes in anteroposterior pelvic diameter (APD), ureteral diameter (UD), and renal function (RF) of the affected upper moiety, assessed using renal ultrasonography and radionuclide imaging, with preoperative measurements serving as the baseline reference. The minimum follow-up duration was 12 months. Surgical success was determined based on fulfillment of all three criteria: resolution or alleviation of clinical symptoms, a reduction in APD and UD, and preserved or improved upper-moiety renal function compared with baseline. **Results:** The final cohort consisted of 52 infants (RALUU, *n* = 28; LUU, *n* = 24). Demographic and clinical profiles were comparable between groups. RALUU was associated with a shorter operative duration than LUU (139.6 ± 16.6 vs. 151.8 ± 21.6 min, *p* = 0.029). Estimated blood loss, time to oral feeding, FLACC pain score, and hospital stay were comparable. Postoperative complications were observed in 2 RALUU patients and 3 LUU patients. One patient in the LUU group developed urine leakage, which was managed conservatively. Postoperative urinary tract infection occurred in 2 patients in each group. No patient required secondary surgery. At a mean follow-up of 26.8 ± 10.4 and 28.1 ± 11.7 months in the RALUU and LUU groups, both groups showed significant reductions in APD and UD, with preserved RF and a modest postoperative increase. **Conclusions:** Both RALUU and LUU were safe and effective for duplicated kidney malformations in infants. RALUU was associated with a shorter operative time, while postoperative recovery, complication rates, and follow-up outcomes were comparable.

## 1. Introduction

Duplicated kidney malformations occur in approximately 0.5–0.8% of children, with female predominance. The condition frequently involves the upper-pole ureter and is often associated with obstructive ureterocele or ectopic ureter. Clinical presentation may include progressive hydronephrosis, urinary incontinence, abdominal pain, or recurrent urinary tract infections (UTIs) [1]. Preservation of renal function has increasingly become a primary goal in the management of duplicated kidney malformations. Ureteroureterostomy (UU) has therefore emerged as an effective reconstructive alternative to heminephroureterectomy in appropriately selected patients.

Open ureteroureterostomy (OUU) remains an established surgical option [2,3,4]. As minimally invasive surgery becomes more widely used, LUU has emerged as an alternative approach with favorable outcomes [5,6,7,8]. More recently, RALUU has also been introduced as a minimally invasive reconstructive option for children with duplicated kidney malformations [9,10].

However, previous clinical studies have evaluated pediatric duplicated kidney malformations across broad age ranges, with limited attention to infants as a distinct surgical population [11,12]. Recent reports have demonstrated the feasibility and safety of RALUU. Villanueva et al. compared 8 infants who underwent RALUU and 12 who underwent OUU and showed that RALUU was safe and effective, with operative times similar to those of OUU performed through a Pfannenstiel incision [13]. Suarez Arbelaez et al. further compared robotic and open reconstruction in infants, including 12 RALUUs and 9 OUUs, and reported that robotic surgery was feasible and safe [14]. Nevertheless, available studies have generally involved relatively small cohorts and mainly compared robotic surgery with open surgery rather than conventional laparoscopy.

In infants, the limited pelvic and retroperitoneal working space, small ureteral caliber, and fragile tissues may increase the technical demands of precise ureteral dissection and intracorporeal anastomosis. Therefore, outcomes derived from older pediatric cohorts may not be directly generalizable to infants. Direct comparative data evaluating RALUU and LUU specifically in infants remain limited. Therefore, we conducted a comparative analysis to assess perioperative recovery, operative efficiency, and follow-up outcomes between RALUU and LUU in infants. We hypothesized that RALUU would achieve perioperative and functional outcomes comparable to those of LUU in infants with duplicated kidney malformations.

## 2. Materials and Methods

We retrospectively reviewed infants who underwent RALUU or LUU between May 2021 and April 2025. Preoperative evaluations included ultrasonography, magnetic resonance urography (MRU), and voiding cystourethrography (VCUG), which confirmed upper pole ureteral abnormalities such as obstructive ureterocele or ectopic ureteral orifice, with no vesicoureteral reflux (VUR) or ureteral stricture in the ipsilateral lower pole ureter. DTPA renography was performed in each patient to evaluate the renal function (RF) of the upper pole moiety.

### 2.1. Inclusion and Exclusion Criteria

Inclusion criteria were as follows: (1) diagnosis of duplicated kidney malformations involving the upper moiety, such as obstructive ureterocele or ectopic ureteral orifice; (2) presence of surgical indications, including progressive upper-moiety hydronephrosis or recurrent UTI not adequately controlled with antibiotic therapy; and (3) preserved differential function of the affected upper moiety, defined as >10% of global RF on radionuclide renal scintigraphy.

**Figure 1:**
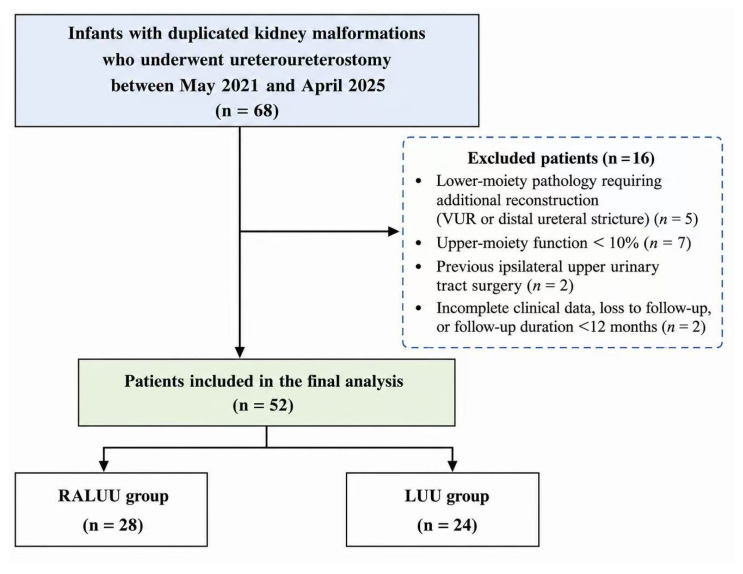
Flowchart of patient selection.

Exclusion criteria were as follows: (1) associated lower-moiety ureteral lesions, including VUR or distal ureteral stricture; (2) severely impaired or non-functional upper moiety (<10% differential function on radionuclide renal scintigraphy); (3) previous ipsilateral upper urinary tract surgery; (4) incomplete clinical data; or (5) follow-up duration shorter than 12 months. A flowchart illustrating patient selection is shown in Figure 1.

Allocation to RALUU or LUU was not based on disease severity or anatomical complexity. Treatment modality was primarily determined by robotic platform availability on the scheduled operative date and parental preference following standardized counseling regarding risks and benefits of each approach. All procedures were performed by an experienced surgeon. Prior to the initiation of this study, the primary surgeon had completed 92 pediatric laparoscopic upper urinary tract reconstructive procedures and 65 robot-assisted upper urinary tract reconstructive procedures, including 22 LUU and 16 RALUU, respectively.

The Ethics Committee of our organization approved this study (2024-IRB-0369-P-01).

### 2.2. Surgical Technique

**Figure 2:**
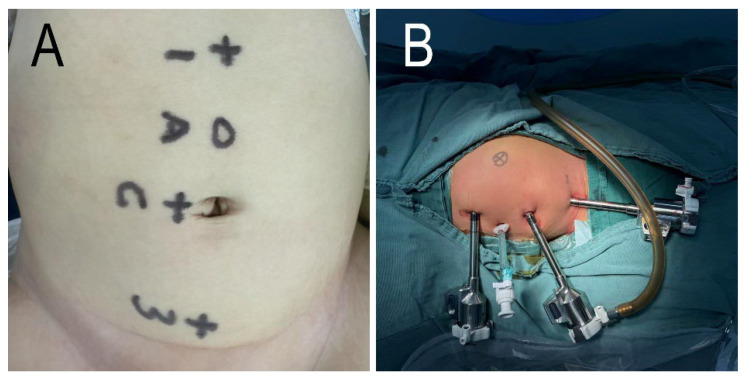
Trocar positions in RALUU. (**A**) Preoperative abdominal surface markings indicating the planned camera port (C), robotic working ports (1 and 3), and assistant port (A). (**B**) Intraoperative view showing the final trocar configuration after port placement, including one camera port and two robotic working ports.

RALUU was performed using the da Vinci Xi Surgical System (Intuitive Surgical Inc., Sunnyvale, CA, USA). Following induction of general anesthesia and endotracheal intubation, the patient was positioned supine with elevation of the affected side. In RALUU, pneumoperitoneum was created via an 8 mm umbilical robotic camera trocar. Two 8 mm working ports were positioned—one subxiphoid near the midline and the other along the Pfannenstiel line inferior to the umbilicus. An extra 5 mm port was created between the camera and upper ports (Figure 2).

Adequate exposure of the operative field was achieved under laparoscopy. If necessary, the colon was mobilized medially to improve exposure. The peritoneum was then opened in an avascular plane, allowing exposure of the upper-moiety ureter, which was carefully dissected and clearly distinguished from the normal lower-moiety ureter.

The distal ureter was mobilized toward the common ureteral sheath, ligated and excised. The dilated ureter was then transected, and the ureteral diameter was assessed. When the diameter exceeded 1.5 cm, appropriate trimming and tapering were performed. Subsequently, the recipient ureter was opened longitudinally to match the donor ureter diameter.

**Figure 3:**
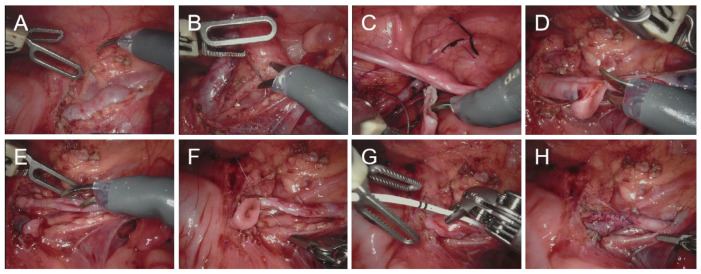
Surgical steps of RALUU. (**A**) The dilated upper moiety ureter was exposed after lateral peritoneal incision. (**B**) The ureters of the upper and lower moieties were carefully separated. (**C**) The distal upper ureter was ligated and transected above the common sheath. (**D**) The redundant segment was excised and obliquely spatulated. (**E**) The recipient ureter was opened longitudinally. (**F**) The donor ureter was obliquely spatulated and anastomosed end-to-side to the recipient ureter. (**G**) A JJ stent was placed through the anastomosis. (**H**) The completed anastomosis demonstrated a Y-shaped configuration.

UU was performed between the transected end of the dilated ureter and the lateral wall of the recipient ureter, ensuring a tension-free and non-twisted Y-shaped configuration. A double-J ureteral stent was placed through the trocar. Finally, the peritoneum was repaired, and the detailed surgical steps are shown in Figure 3A–H.

For LUU, one 5 mm camera port and two 3 mm trocars were established, positioned at the suprainguinal and subxiphoid regions. The operative procedure followed steps comparable to RALUU.

### 2.3. Data Comparison and Follow-Up

Perioperative outcomes assessed in this study included operative duration, blood loss, time to oral intake, FLACC pain score on postoperative day 1, length of hospitalization, and postoperative complications. Docking time and console time were also documented for patients undergoing RALUU. Operative time was measured from skin incision to wound closure. In the RALUU group, docking time referred to the interval required for robotic system setup after trocar placement, and console time represented the duration of robotic instrument manipulation. Postoperative complications were classified using the Clavien–Dindo system. The minimum follow-up duration was 12 months. Follow-up assessments included renal ultrasound at 1, 3, 6, and 12 months after surgery and renal scintigraphy at 12 months after surgery. Imaging and functional outcomes included APD, UD, and RF of the pathological upper pole unit. RF was assessed for the affected moiety in a duplicated collecting system rather than for the whole kidney. A postoperative change within ±2% compared with baseline was defined as preserved renal function, an increase of more than 2% as improved renal function, and a decrease of more than 2% as renal functional deterioration. Double-J stents were generally removed 4–6 weeks after surgery according to the patient’s postoperative condition and imaging findings. For bilateral cases, both affected renal units were included separately in the radiological and functional outcome analyses. Other perioperative parameters and clinical outcomes were analyzed on a per-patient basis. Surgical success was based on symptom relief, together with ultrasound evidence of reduced hydronephrosis and ureteral dilation, with preserved or improved renal function.

### 2.4. Statistical Methods

SPSS Statistics for Windows, Version 27.0 (IBM Corp., Armonk, NY, USA) was used for statistical analysis. Normally distributed continuous data are shown as mean ± SD with 95% confidence intervals, whereas skewed data are expressed as median and interquartile range. Continuous and categorical variables were analyzed using appropriate statistical tests according to data distribution and variable type. Statistical significance was set at *p* < 0.05.

## 3. Results

### 3.1. Patient Characteristics

The final cohort consisted of 52 infants (RALUU, *n* = 28; LUU, *n* = 24). Demographic and clinical profiles are shown in Table 1. The two groups were comparable in age, weight, sex, laterality, pathological type, and surgical indications.

### 3.2. Intraoperative Outcomes

All procedures were completed successfully. RALUU was associated with a shorter operative duration than LUU (139.6 ± 16.6 min, 95% CI: 133.1–146.0 vs. 151.8 ± 21.6 min, 95% CI: 142.7–160.9; *p* = 0.029). In the RALUU group, the mean docking time was 14.5 ± 3.8 min (95% CI: 13.8–16.2), and the mean console time was 111.2 ± 15.6 min (95% CI: 108.2–120.1). Estimated blood loss was comparable. These results are summarized in Table 1.

### 3.3. Postoperative Recovery and Complications

Postoperative recovery was comparable. The time to oral feeding, FLACC pain score, and hospital stay showed no significant differences. Complications were recorded in 2 RALUU patients (7.1%) and 3 LUU patients (12.5%) (*p* = 0.652). One patient in the LUU group developed urine leakage, which was managed conservatively. UTI was observed in 2 patients in each group. No patient required secondary surgery. The postoperative outcomes are shown in Table 1.

### 3.4. Follow-Up Outcomes

At a mean follow-up of 26.8 ± 10.4 and 28.1 ± 11.7 months in the RALUU and LUU groups, APD and UD significantly decreased after surgery in both groups. RF was preserved, with a modest increase. Intergroup comparisons showed no significant differences in preoperative, postoperative, or change values for APD, UD, or RF. These results are summarized in Table 2.

## 4. Discussion

In the present study, we compared perioperative and follow-up outcomes between RALUU and LUU in infants with duplicated kidney malformations. Both approaches were successfully completed. The two groups had comparable baseline characteristics, pathological types, and surgical indications, indicating acceptable baseline comparability between groups.

The main difference observed in this study was operative time. RALUU resulted in a shorter operative time than LUU. This finding is consistent with previous reports. Chertin and Yu reported that RALUU was associated with a significantly shorter operative time than laparoscopic ipsilateral ureteroureterostomy in children [11,12]. Although their cohort included children older and heavier than the infants in the present study, the direction of the finding was similar. In addition, evidence from other infant upper urinary tract reconstructions also supports this trend. Shu and Sun reported a significantly shorter operative time for robot-assisted laparoscopic pyeloplasty compared with laparoscopic pyeloplasty in infants and young children [15,16]. Nevertheless, the clinical relevance of the reduction in operative time should be interpreted with caution, given the retrospective non-randomized design of this study and the potential influence of patient selection and surgeon experience.

These findings suggest that the shorter operative time in the RALUU group may be related to improved efficiency in selected intracorporeal reconstructive steps. In infant UU, accurate identification of the upper- and lower-pole ureters, creation of a well-matched anastomotic opening, and completion of a tension-free anastomosis are essential. The robotic platform may assist with these technically demanding steps.

Learning curve effects should also be considered when interpreting operative time. Previous studies have suggested that robotic upper urinary tract reconstruction in children may reach technical proficiency after a relatively small number of cases. Andolfi et al. reported that laparoscopic pyeloplasty reached a plateau after approximately 18 cases, whereas robot-assisted laparoscopic pyeloplasty achieved an initial phase of proficiency after approximately 13 cases [17]. Zhou et al. further showed that the learning curve for robotic pyeloplasty entered a relatively stable phase after approximately 11 cases for console time and after approximately 19 cases when single-suture time was used as the performance metric [18]. In the present study, all cases were managed by the same experienced pediatric urologic surgeon. Therefore, although learning curve effects cannot be completely excluded, their influence on the observed perioperative outcomes was likely limited.

Although operative duration was shorter in the RALUU group, other perioperative recovery parameters were similar. Estimated blood loss, time to oral feeding, postoperative FLACC pain score, and hospital stay showed no significant differences. These findings suggest that the shorter operative duration observed in the RALUU group did not correspond to measurable differences in early postoperative recovery parameters in this cohort. Therefore, the shorter operative time observed in the RALUU group may reflect improved technical efficiency during ureteral reconstruction, whereas its impact on early postoperative recovery appears limited based on the current data.

With respect to complications, the overall incidence was comparable. Two patients in each cohort developed postoperative UTI requiring antibiotic therapy (Clavien–Dindo grade II). In addition, one patient in the LUU group experienced urine leakage, which was managed conservatively. Importantly, no secondary surgical intervention was required in either group. These findings suggest that both approaches are safe when performed in experienced hands.

During follow-up, both groups demonstrated significant reductions in APD and UD, together with preservation of renal function. The mean postoperative increase in DRF was approximately 2% in both groups; however, this degree of change may fall within the inherent variability of DTPA renography measurements and should therefore be interpreted with caution.

The functional benefit of surgery may therefore be interpreted as preservation of renal function, with a possible mild improvement. Taken together with the significant radiological improvement and the absence of reoperation during follow-up, these findings suggest that both surgical approaches effectively relieved obstruction and maintained stable renal function.

Robotic surgery generally involves additional costs related to equipment acquisition, maintenance, and disposable instruments, resulting in higher overall healthcare expenditures than conventional laparoscopy [19]. In the present study, although RALUU was associated with a shorter operative time, the two groups demonstrated largely comparable perioperative recovery and follow-up outcomes. Therefore, from a cost-effectiveness perspective, conventional laparoscopy remains a safe, effective, and cost-effective option in centers with extensive laparoscopic experience.

Looking ahead, the two approaches are more likely to play complementary rather than competing roles. Robotic surgery may be particularly advantageous for complex reconstructive cases, especially in high-volume centers with established robotic platforms and relevant expertise. In contrast, conventional laparoscopy is likely to remain an important treatment option because of its greater accessibility and lower cost [19]. Therefore, the choice of surgical approach should be individualized according to anatomical complexity, surgeon experience, robotic platform availability, healthcare costs, and family preference.

Based on our experience, several technical considerations are important for successful ureteroureterostomy. First, meticulous dissection is required to clearly distinguish the ureters of the upper and lower moieties. The ureter should be adequately mobilized to ensure a tension-free anastomosis, while vascular injury should be carefully avoided. Second, adequate oblique spatulation of the donor ureter, together with a diameter-matched longitudinal incision on the lateral wall of the recipient ureter, helps create a wide and well-aligned anastomosis. Finally, suturing the posterior wall first, followed by appropriate stent placement and completion of the anterior wall, facilitates precise intracorporeal suturing and ensures a tension-free, non-twisted Y-shaped reconstruction, thereby maintaining unobstructed urinary drainage.

Certain limitations of this study should be recognized. First, this investigation was based on a relatively small retrospective cohort. Second, patient allocation was influenced by robot availability and family preference rather than randomization, which may have introduced selection bias. Because propensity score matching and multivariable adjustment were not performed, residual confounding from unmeasured factors may have persisted. Therefore, the observed differences between groups may not be entirely attributable to the surgical approach itself. Extended follow-up is required to determine long-term efficacy.

Overall, RALUU and LUU were feasible minimally invasive options for duplicated kidney malformations in infants. RALUU shortened operative time, while other perioperative and follow-up outcomes were comparable. Additional prospective studies comparing RALUU and LUU in infant UU are warranted.

## Figures and Tables

**Table 1:** Baseline and Perioperative Data.

Variable	RALUU (*n* = 28)	LUU (*n* = 24)	*p* Value
Age (months)	7.8 ± 2.4 (6.8–8.7)	7.3 ± 2.9 (6.1–8.5)	0.499
Weight (kg)	8.1 ± 1.5 (7.5–8.7)	8.2 ± 1.9 (7.4–9.0)	0.833
Gender, *n* (%)			0.816
Male	9 (32.1%)	7 (29.2%)	
Female	19 (67.9%)	17 (70.8%)	
Laterality, *n* (%)			0.832
Left	14 (50%)	14 (58.3%)	
Right	11 (39.3%)	8 (33.3%)	
Bilateral	3 (10.7%)	2 (8.3%)	
Type of Pathology, *n* (%)			0.815
Ureteral ectopia	24 (85.7%)	22 (91.7%)	
Ureterocele	4 (14.3%)	2 (8.3%)	
Surgical indication, *n* (%)			0.532
Hydronephrosis	14 (50%)	9 (37.5%)	
UTI	14 (50%)	15 (62.5%)	
Operative time (min)	139.6 ± 16.6 (133.1–146.0)	151.8 ± 21.6 (142.7–160.9)	0.029
Docking time (min)	14.5 ± 3.8 (13.8–16.2)	—	
Console time (min)	111.2 ± 15.6 (108.2–120.1)	—	
Estimated blood loss (ml)	10.8 ± 4.5 (9.1–12.6)	12.1 ± 5.0 (10.0–14.3)	0.329
Oral feeding time (h)	8.5 ± 2.2 (7.7–9.4)	8.9 ± 2.4 (7.8–9.9)	0.535
FLACC pain score	2.1 ± 1.2 (1.6–2.5)	2.4 ± 1.1 (2.0–2.9)	0.207
Hospital stay (days)	4.8 ± 1.1 (4.3–5.2)	5.2 ± 1.2 (4.7–5.7)	0.216
Complications, (Clavien–Dindo, *n*), *n* (%)	2 (7.1%)	3 (12.5%)	0.652
Urine leakage (Grade I)	0	1 (4.2%)	
UTI (Grade II)	2 (7.1%)	2 (8.3%)	
Secondary surgery (Grade III)	0	0	

**Table 2:** APD, UD, and RF of affected upper moiety units in the RALUU and LUU groups.

Variable	RALUU (*n* = 31 Units)	LUU (*n* = 26 Units)	*p* Value
Preoperative APD (mm)	20.4 ± 7.3 (17.7–23.1)	20.7 ± 6.6 (18.0–23.4)	0.881
Postoperative APD (mm)	7.5 ± 3.5 (6.2–8.8)	7.8 ± 2.3 (6.9–8.7)	0.704
Change in APD (mm)	12.9 ± 4.8 (11.1–14.7)	12.9 ± 5.5 (10.7–15.1)	0.990
Preoperative UD (mm)	11.8 ± 5.0 (9.9–13.6)	11.7 ± 5.4 (9.5–13.9)	0.975
Postoperative UD (mm)	4.9 ± 1.7 (4.3–5.6)	5.0 ± 2.7 (3.9–6.1)	0.890
Change in UD (mm)	6.8 ± 4.0 (5.4–8.3)	6.7 ± 3.8 (5.2–8.2)	0.901
Preoperative RF (%)	14.5 ± 2.6 (13.5–15.5)	14.1 ± 3.0 (12.9–15.3)	0.590
Postoperative RF (%)	16.7 ± 3.4 (15.4–17.9)	16.5 ± 3.0 (15.3–17.7)	0.830
Change in DRF (%)	2.2 ± 1.2 (1.8–2.6)	2.4 ± 0.9 (2.0–2.8)	0.432

## Data Availability

The study data are available from the corresponding author upon request.
